# Designing, Implementing, and Evaluating a Home-Based, Multidisciplinary, Family-Centered Pediatric Obesity Intervention: The ProxOb Program

**DOI:** 10.3390/children9050737

**Published:** 2022-05-17

**Authors:** Magalie Miolanne, Céline Lambert, Julie Masurier, Charlotte Cardenoux, Alicia Fillion, Sarah Beraud, Chloé Desblés, Amélie Rigal, Elodie Védrine, Carla Dalmais, Bernadette Da Silva, Elisabeth De L’Eprevier, Juliette Hazart, Jean-Philippe Chaput, Vicky Drapeau, Bruno Pereira, Grace O’Malley, David Thivel, Yves Boirie

**Affiliations:** 1 Department of Human Nutrition, CHU Gabriel Montpied, 63000 Clermont-Ferrand, France; mdebouit@chu-clermontferrand.fr (M.M.); yboirie@chu-clermontferrand.fr (Y.B.); 2 Auvergne Obesity Specialized Center CALORIS, 63000 Clermont-Ferrand, France; julie.masurier-chateau@ugecam.assurance-maladie.fr (J.M.); c.cardenoux@centremedicalinfantile.com (C.C.); sarah.beraud@gmail.com (S.B.); chloe.desbles@reppop-lyrra.fr (C.D.); arigal@chu-clermontferrand.fr (A.R.); evedrine@chu-clermontferrand.fr (E.V.); carladalmais@gmail.com (C.D.); caloris@chu-clermontferrand.fr (B.D.S.); 3 Unit of Biostatistics (DRCI), Clermont-Ferrand University Hospital, 63000 Clermont-Ferrand, France; clambert@chu-clermontferrand.fr (C.L.); edeleprevier@chu-clermontferrand.fr (E.D.L.); bpereira@chu-clermontferrand.fr (B.P.); 4 Laboratory of the Metabolic Adaptations to Exercise under Physiological and Pathological Conditions (AME2P), Clermont Auvergne University, 63000 Clermont-Ferrand, France; fillonalicia@gmail.com; 5 French National Observatory for Physical Activity and Sedentary Behaviors (ONAPS), 63000 Clermont-Ferrand, France; 6 Service de Santé Publique, CHU de Clermont-Ferrand, CEDEX 1, 63058 Clermont-Ferrand, France; juliette.hazart@hotmail.fr; 7 Healthy Active Living and Obesity Research Group, Children’s Hospital of Eastern Ontario Research Institute, Ottawa, ON K1A 0A1, Canada; jpchaput@cheo.on.ca; 8 Centre NUTRISS, Department of Physical Education, Institute of Nutrition and Functional Foods, Université Laval, Québec City, QC G1A 0A2, Canada; vicky.drapeau@fse.ulaval.ca; 9 School of Physiotherapy, Division of Population Health Sciences, RCSI University of Medicine and Health Sciences, D02 YN77 Dublin, Ireland; graceomalley@rcsi.ie; 10 Child and Adolescent Weight Management Service, Children’s Health Ireland at Temple Street, D02 YN77 Dublin, Ireland; 11 International Research Chair Health in Motion, Clermont Auvergne University Foundation, 63000 Clermont-Ferrand, France; 12 INRA, UMR 1019, CRNH Auvergne, 63000 Clermont-Ferrand, France

**Keywords:** family-based, home setting, pediatric obesity, prevention, treatment

## Abstract

Although family-based interventions have been suggested as promising approaches for preventing and treating pediatric obesity, available studies failed to include the whole family in its own natural environment and routine. This paper aims to detail the development, implementation, and evaluation phases of the ProxOb home-based, family-centered program and present its feasibility and early results. ProxOb provides families with a 6-month multidisciplinary, home-based, and family-centered intervention followed by an 18-month maintenance phase. A global psychosocial, clinical, and behavior evaluation was conducted at baseline (T0) at the end of the 6-month intervention (T1) and after the 18-month maintenance phase (T2). A total of 130 families with at least one child with obesity completed the ProxOb program so far, and more than 90% of them also presented at least one parent with overweight or obesity. Being part of a single-parent family seemed to increase the chance of completing the intervention (63.0% vs. 33.3% in the drop-outers subgroup, *p* = 0.03). The BMI z-score for children with obesity (T0 = 4.38 ± 1.05; T1 = 4.06 ± 1.07; T2 = 4.29 ± 1.12) significantly decreased between T0 and T1, followed by weight regain at T2. ProxOb proposes a feasible and replicable real-life approach to address childhood obesity while involving the children’s family.

## 1. Introduction

Children and adolescents are facing alarming rates of overweight and obesity, leading to numerous metabolic, functional, academic, and/or psychosocial complications among others. Since children with obesity are at higher risk of having obesity during adolescence and adulthood [1], there is a clear need for effective preventive and treatment strategies from an early age.

While school-based interventions have been shown to be somewhat effective in preventing the development of pediatric obesity [2,3], some experts also underline the importance of employing family-based approaches to improve the efficacy of body weight management strategies among children [4,5]. Indeed, the family unit has been shown to have a powerful impact on the development and maintenance of children’s activity, dietary and leisure behaviors, strongly suggesting that parents, siblings, and caregivers should be involved in interventions elaborated to enhance healthy eating and healthy, active living in children and youth [6,7].

Data suggest that family-based programs combining physical activity and dietary interventions and specific behavioral treatments can be effective in slowing or reducing overweight and/or obesity in children below 12 years of age [8,9,10]. Very recently, Arnason and colleagues published a systematic review and meta-analysis questioning the necessary characteristics for successful family-based interventions addressing childhood obesity [11]. Based on their analysis of the 34 included studies that reported body mass index z-scores (z-BMI), the authors concluded that successful interventions need to last between 6 to 12 months and should include education and training aimed at parents and health practitioners; a multidisciplinary approach with dietary, physical activity, psychological, and social education and support; counseling on parenting in the context of pediatric obesity; and the use of online and electronic (e-tools) supports [11]. Another major objective of these family interventions should also be to reach underprivileged families who do not readily engage with the health service.

While such family-based interventions seem promising, most of the studies conducted so far have been implemented in clinical, community, or school settings [9,12], failing to fully include the whole family in its usual environment and routine. Although Varagiannis and collaborators recently proposed the “4 your family” program targeting 8–12-year-old children with overweight or obesity, families in their “home-based” intervention arm only followed an online program at home [13]. In 2016, Appelhans and colleagues also conducted a meta-analysis based on 15 interventions qualified as “home-based” family programs targeting pediatric obesity [14]. However, according to their analysis, only some clinical evaluations were performed at home, and the main intervention mainly consisted of phone calls with a maximum of one physical visit at the families’ accommodation. Indeed, the core interventions were all conducted in public institutions, and most of the time, with several families at a time [14]. Moreover, the authors concluded that there was a high level of heterogeneity when it came to both the designs and reported results of the included trials [14]. Interestingly, Kinlin and colleagues recently proposed an assessment of the feasibility and acceptability of their STOMP-EY family-based intervention, in which home visits from public health nurses were performed in addition to the parent-only group sessions [15]. According to their results, such family-based programs that include home visits face several barriers, such as the lack of relative priority and perceived patient need, lack of tailoring to individual patient needs, a poor parental motivation to engage in group sessions, and the challenges related to the scheduling and delivery of group sessions [15].

In that context, and in line with the objectives of the French National Obesity Plan, the Auvergne Regional Obesity Specialized Center developed the ProxOb program (a home-based, family-centered intervention addressing childhood obesity). This article presents the development, implementation, and evaluation phase preliminary results.

## 2. Materials and Methods

### 2.1. Origins and Development

As part of its National Obesity Plan launched in 2010, the French Minister of Health established 37 Specialized Obesity Centers (CSO) with the mission to structure, coordinate, and develop the prevention and treatment of obesity and related scientific activities at a regional level. In that context, the Auvergne territory CSO, called “CALORIS” (Centre Auvergnat de L’Obésité and de ses RIsques en Santé) centered its activities on the development of innovative multidisciplinary interventions targeting pediatric obesity with a focus on providing access to care for all in need of intervention.

**Figure 1:**
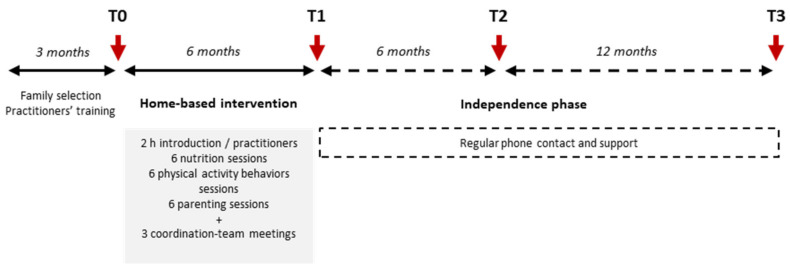
Design of the ProxOb program. From ProxOb I to IV, the program stopped at T2.

After a careful evaluation of the regional situation and the scientific and clinical literature, CALORIS developed a pilot program in 2015 to propose home-based interventions combining physical activity education, nutritional education, and psychological and parenting support to families concerned with pediatric obesity: the ProxOb program. The ProxOb program aims to provide families with personalized interventions conducted by specialists according to the following steps: (i) specific training programs for health practitioners; (ii) screening of families interested in participating; (iii) baseline clinical evaluation of each family member and evaluation of the family situation (T0); (iv) delivery of a 6-month multidisciplinary, home-based, and family-centered intervention; (v) a second evaluation phase at the end of the 6-month intervention (T1); (vi) an 18-month maintenance phase with regular phone support (with an evaluation session after the first 6 months of independence (12 months from baseline—T2); and (vii) a final evaluation at the end of the overall 18 months of the independence phase (T3). From the first ProxOb campaign (ProxOb I) to the fourth (ProxOb IV), the program stopped at T2, and the following 12 months of independence phase and the T3 assessment point were added from ProxOb V (5th ProxOb cohort). Figure 1 illustrates the design of the ProxOb program.

### 2.2. Implementation

#### 2.2.1. Specific Training of the Practitioners

The CALORIS team, together with specialized trainers from the National Association for Therapeutic Education, created a specific training curriculum leading to an official qualification, particularly focusing on obesity. All the practitioners (as detailed below) involved in the ProxOb program followed a 40 h course describing the etiology of obesity and its prevention and treatment and the elements of a home-based and family-centered intervention and detailing all the specific tools and activities specifically developed for the interventions. The training phase is a highlight of the program since the presence and quality of training have been identified in the literature as an important factor contributing to the success of family-based obesity intervention [11].

#### 2.2.2. Participating Families

To take part in the ProxOb program, the families have to have: (i) at least one child under 18 years of age who is living with overweight or obesity and (ii) a child living with at least one parent or a family with a child under 6 years (before adiposity rebound) where both parents have obesity (BMI ≥ 30 kg/m²). In addition, the families need to present with at least one of the following criteria: (i) being classified with a precarious social situation (according to the EPICES score [16]); (ii) living in an area with reduced health care access; (iii) without any access to health care; or (iv) unsuccessful previous obesity intervention. In addition to these criteria, the candidate families had to report sufficient availability to complete the different project evaluations and to welcome the practitioners into their home during the intervention.

Families with at least one parent presenting with a BMI above 45 kg/m^2^ without individual treatment or refusing to follow recommended treatment were excluded. Similarly, families whose child suffered from a syndromic cause of obesity without designated healthcare and who presented with mental and cognitive disorders were not included. Importantly, the overweight and obesity status in children were calculated using the CALIMCO software developed by the French National Nutrition and Health Plan (PNSS), using French adapted curves.

#### 2.2.3. Intervention

The ProxOb program rests on an educational approach (therapeutic education intervention) where families received adapted and individualized counseling (there was no prescribed nutritional intervention nor physical training). Each family was assigned to three practitioners composed of one physical activity and health educator, one nutrition and dietetics specialist, and one psychologist and/or social worker. Each practitioner separately visited the home for an introductory 2 h visit, and thereafter, each one visited the family over 6 months for six sessions. The three practitioners were in constant contact concerning the families, and a multidisciplinary meeting with the pediatrician was held on three occasions during the 6-month period. Importantly, the usual general practitioners of the families were all involved in the process and were constantly informed of their evaluation. The educational sessions delivered by the practitioners addressed the following main topics: 

Physical activity and health: Definitions and benefits of physical activity and sedentary behaviors; “What do we mean by structured sport?”; “How to be active at home?”; “How and where to be active in my neighborhood and region?”.

Nutrition and dietetics: Definition and implication of weight loss and dietary restrictions; “What is a healthy and balanced diet?”; presentation of the main food families; determination and detection of appetite sensations; “How to anticipate and prepare a meal?”; “How to handle snacking and food intake outside meals?”; Cooking lessons and tips.

Psychological approach: These sessions were mainly focused on “parenting in the context of pediatric obesity and covered the following topics”: “How to communicate within the family sphere?”; “How to handle our kids and the overall family sleep needs and rhythm?”; “How to handle screen time at the family level?”; definition and identification of our emotions and senses; the need to suppress the “We/I don’t have enough time” from our daily routine; definition, identification, and management of stress.

### 2.3. Evaluations

As detailed in Figure 1, clinical and behavioral evaluations were performed on four occasions: at baseline (T0) and after the 6-month home-based intervention (T1); after the first 6 months of the maintenance phase (T2); and at the end of the maintenance phase (T3). Tables S1 and S2 (Supplementary Materials) detail the outcome measurements and questionnaires filled by the parents and children, respectively; the time points of assessment; and the evaluation of the intervention from ProxOb I (2015–2016) to ProxOb V (2020–2021). As detailed, some of the methods used changed over time based on the advantages or limitations experienced during the previous editions of the program. On top of these evaluations, qualitative interviews were briefly performed when possible with families withdrawing from the intervention to identify reasons for dropout.

### 2.4. Statistical Considerations

Statistical analysis was performed using STATA (version 15; StataCorp, College Station, TX, USA). All tests were two-sided, with a Type I error set at 0.05. Categorical data were expressed as frequencies and associated percentages and continuous data as mean ± standard deviation or median (1st quartile; 3rd quartile), according to statistical distribution. The Gaussian distribution was checked with the Shapiro–Wilk test and/or by histogram. Families were compared based on whether they completed the program using the chi-squared test or Fisher’s exact test for categorical variables and Mann–Whitney test for quantitative variables. The evolution of z-BMI (for children) and BMI (for parents) over time (T0, T1, T2) was evaluated by linear mixed-effects models (T3 was not yet available at the time of the analysis), with ProxOb edition, with family and subject considered as random effects. Residuals normality of all models was studied. Characteristics of the “completers” and “drop-outers” were also compared, with completers being determined as families who properly completed the entire intervention up to T1 (Figure 1).

## 3. Results

### 3.1. Descriptive Characteristics of the Families

**Figure 2:**
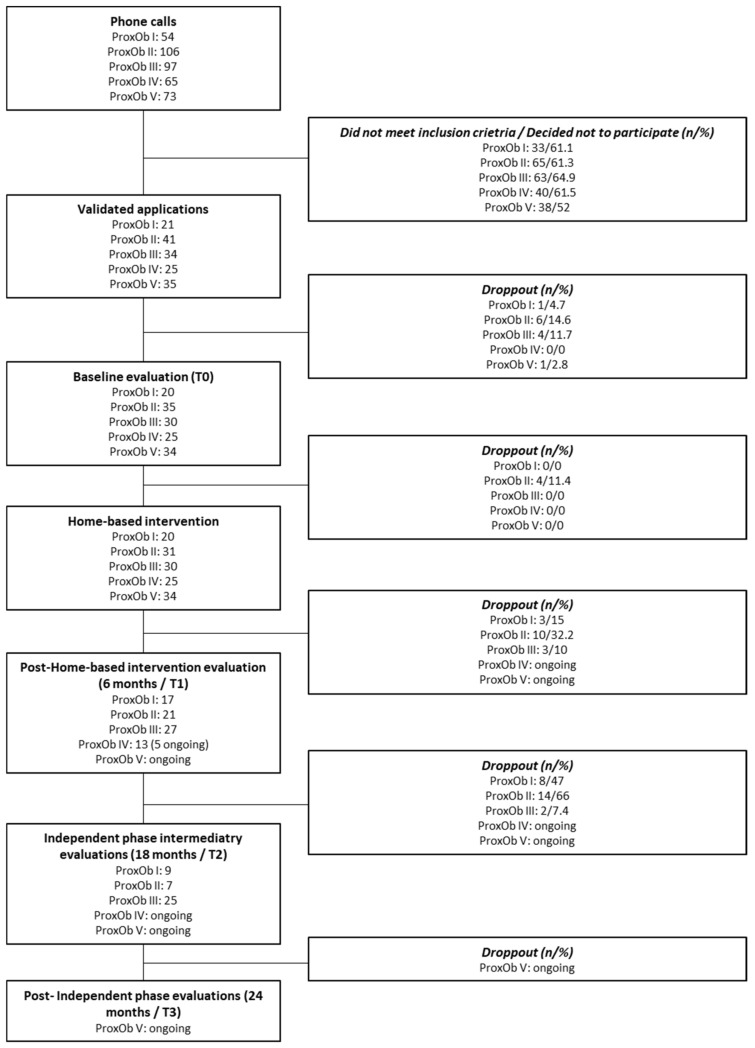
Flow-chart of the families’ inclusion. T0, baseline; T1, end home-based intervention; T2, after 6 months of individualization.

Figure 2 presents the flow-chart of the successive annual ProxOb program. Proxim IV post-home-based intervention evaluation and ProxOb V intervention is ongoing. Table 1 details the characteristics of the enrolled families as well as the weight status of the family members. While to be included, families have to present at least one child with overweight or obesity, between 90.9% to 100% of the enrolled families also present at least one parent with overweight and/or obesity. Overall, 50 to 73.7% of the enrolled families have at least one parent in a precarious economic situation (based on the EPICE score).

### 3.2. Preliminary Results

As shown in Table 2, being part of a single-parent family seems to increase the chance of completing the intervention (63.0% vs. 33.3% in the drop-outers subgroup, *p* = 0.03), which is in line with the results of the qualitative evaluation of the intervention with families declaring it difficult to mobilize both parents (particularly fathers) for practical and motivational reasons. The number of family members, the number of children per family or the precarious situation of the family do not seem to explain the adherence to the intervention. Table 2 presents the main comparisons between completers and drop-outers. A more qualitative approach revealed some reasons for dropout, the main one being family house move; pregnancies; difficulties to engage both parents (mainly fathers), and difficulties in finding time for the home-based interventions. Importantly, the lower rate of dropouts from ProxOb III can be explained by the introduction of a motivational evaluation of the candidate families to improve their selection and decrease the risk of dropout.

**Figure 3:**
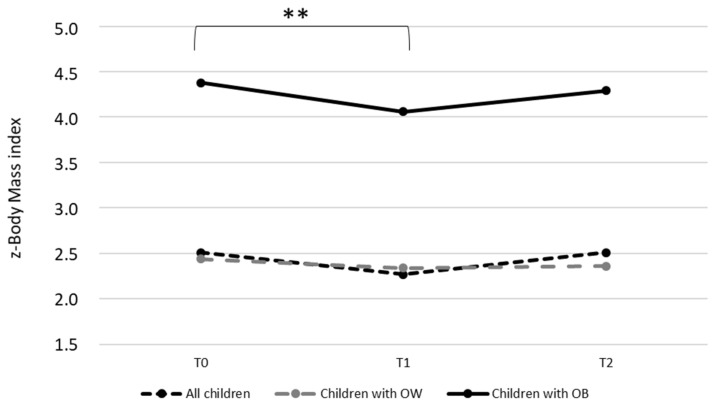
Evaluation of z-body mass index between T0 and T2. OB, obese; OW, overweight; T0, baseline; T1, end home-based intervention; T2, after 6 months of intervention. Statistics were performed on the total sample (ProxOb II to IV); ** *p* = 0.008. Means and standard deviations are detailed in Section 3.

As illustrated in Figure 3, while the z-BMI of the whole sample and of children with overweight did not change between T0 and T2 (all children: T0 = 2.51 ± 2.06; T1 = 2.27 ± 1.89; T2 = 2.51 ± 1.96; children with overweight: T0 = 2.44 ± 0.44; T1 = 2.34 ± 0.46; T2 = 2.36 ± 0.50), there is a significant reduction of z-BMI between T0 and T1 (*p* = 0.008) for children with obesity (T0 = 4.38 ± 1.05; T1 = 4.06 ± 1.07; T2 = 4.29 ± 1.12). The BMI of parents (whole sample and for parents with overweight and obesity separately) did not change between T0 and T2 (data not shown).

## 4. Discussion

While the prevalence of pediatric overweight and obesity remains alarming, there is a clear need for innovative and effective prevention and treatment strategies. On top of inpatient clinical interventions and school-based programs that have been shown somewhat effective in preventing and reducing pediatric obesity, the development of family approaches has been encouraged [4,8], suggesting that parents should be involved to effectively help the development and maintenance of children’s healthy movement and dietary and leisure behaviors [6,7]. While several “family-based interventions” have been developed, showing effective short-term results on children’s z-BMI [8,9,10], none of them considered the family environment and home setting. In their recent systematic review, Arnasan and colleagues observed that 47% of the included studies (*n* = 40) performed their evaluations and intervention in school settings, with the rest being conducted in community and health-care settings [11]. Only Appelhans et al. intervened at home but only for some measurements and not during their intervention [14]. Similarly, Varagannis et al. performed a family intervention using a home-based online program, missing the proper inclusion of the home setting in their approach [13].

As part of its missions, the Auvergne territory Obesity Specialized Center, CALORIS, and in line with the objectives of the French National Obesity Plan, developed and implemented a home-based, family-centered intervention targeting childhood obesity (the ProxOb program). The ProxOb program includes the main key levers suggested by the literature for effective family-based pediatric obesity interventions. First, ProxOb uses a multidisciplinary approach combining therapeutic education programs in physical activity and movement behaviors, eating habits, psychological supports, and parenting, which was described as essential for such interventions by some authors [17,18,19]. In their study, Janicky et al. (2008) included training sessions to the practitioners and caregivers (two full days + one 6 h boost at mid-intervention), which has also been shown to strengthen the intervention and enhance its effects [17]. In that sense, all the practitioners and professionals involved in the evaluation and intervention during ProxOb follow specific training before the program and attend three collective staff meetings. Importantly, the home-based intervention lasts 6 months, with a subsequent 12-month assisted maintenance phase, in line with the recent meta-analysis from Arnasan et al. (2020) that highlighted the need for at least 6 months of intervention to favor significant effect of the children’s z-BMI [11].

Based on the results of their systematic analysis of the available evidence regarding family-interventions, Arnasan et al. (2020) also clearly pointed out the difficulty to draw strong conclusions regarding their efficacy due to a large methodological heterogeneity between studies, contributing to a 96.3% of variability in the treatment effect [11]. While information is available regarding the design, contents of the interventions, and methods used to evaluate the effect of the programs, we did not find data regarding the characteristics of the families that are usually interested and then involved in such programs and regarding the families that are more willing to complete the interventions. According to the data collected during the last 5 years (as presented in the present tables), the ProxOb program mainly included families suffering from economic difficulty (54.5% to 73.7% of the included families) and almost exclusively included families with at least one parent with obesity (90.9% to 100%). Interestingly, a high proportion were single-parent families (25.6% to 52%). According to our results, the number of family members or of children in the family as well as the presence of at least one parent concerned with obesity were not associated with the completion or dropout rate from the intervention. However, our results suggest that single-parent families are more willing to complete the whole program. This result is in line with the qualitative data obtained during interviews, pointing out the difficulty to get both parents involved, particularly fathers, being expressed by the participants. Importantly, we did not find any association between the completers/dropouts and the edition (year) of the ProxOb program, which indicates that the composition of the practitioner team (that was different between editions) was not a barrier to the success of the intervention. This result reinforces the importance of the staff training and underlines the feasibility of implementing the program in other geographical areas with different teams.

While the present paper provides and details the methods and implementation of a family-centered, home-based, educational pediatric obesity intervention, Figure 3 also suggests its efficacy in the short term when it comes to reducing the z-BMI of children with obesity. According to our results, the ProxOb program significantly reduced the z-BMI of children with obesity after 6 months of home-based intervention. Furthermore, the observed mean reduction of −0.32 of the z-BMI is even higher than the one observed by Arsanan and colleagues in their recent meta-analysis (−0.14 (−0.26; −0.08)) [11]. This encouraging result might be because our program combines the main details specified here again that have been shown to enhance the effect of such approaches as described above. Importantly, however, this significant reduction does not persist during the assisted independence phase. This result, together with the described need for a more constant support for families, led the CALORIS team to propose adaptations for the program’s future edition. First, in line with the results from Arnasan et al. (2020), the home-based intervention phase will be delivered over 12 months (against 6 months actually) in future editions of the ProxOb program, which might potentially strengthen the induced-behavioral modifications [11]. Moreover, a new digital “e-ProxOb” intervention will be now proposed to accompany the intervention phase and will be reinforced during the “maintenance phase”, using a mobile application maintaining the link with the caregivers, reinforcing the support, and prolonging the intervention. This improvement of the ProxOb program by using an e-health application and providing families with regular feedback and support should enhance the effect of the program and its maintenance, as previously suggested [13,17,18]. Finally, while the ProxOb program rests on an educational approach to improve behaviors, future editions should consider the evaluation of some key metabolic parameters to consider the metabolic profile of the children and their families as a potential determining factor for the success of the intervention.

## 5. Conclusions

To conclude, the present paper describes the development, implementation, and evolution of the home-based, family-centered ProxOb program (from one edition to another) and its very first results. Importantly, the ProxOb program, while not initially designed as a clinical research intervention (which explains the detailed evolution of the intervention and methods over time), proposes a feasible and replicable real-life approach to address childhood obesity while involving the children’s family. Although it remains to be scaled up and improved, the ProxOb program has been recently recognized by the French Minister of Health as an effective and promising real-life care program that will now be more deeply evaluated for the next couple of years, with the objective of national dissemination (article 51 French Minister of Health).

## Figures and Tables

**Table 1:** Descriptive characteristics of the families and family members enrolled in the ProxOb programs. Data are presented as number of subjects (percentages), mean ± standard deviation, or median (1st quartile; 3rd quartile).

Families	ProxOb II	ProxOb III	ProxOb IV	ProxOb V
	*n* = 41	*n* = 30	*n* = 25	*n* = 34
Number of people per family	4 (3; 5)	4 (3; 4)	3 (2; 4)	4 (3; 4)
Single-parent family	14 (34.1)	13 (43.3)	13 (52.0)	9 (26.5)
Number of children per family	2 (1; 3)	2 (1; 3)	2 (1; 2)	2 (1; 2)
At least one parent per family with overweight/obesity	40/40 (100.0)	29/30 (96.7)	22/24 (91.7)	30/33 (90.9)
At least one parent with a precarious economic situation	12/22 (54.5)	18/28 (64.3)	14/19 (73.7)	13/26 (50.0)
**Children**	***n* = 87**	***n* = 58**	***n* = 42**	***n* = 64**
*Weight status*				
Underweight	1 (1.2)	0 (0.0)	0 (0.0)	0 (0.0)
Normal	36 (41.4)	14 (24.1)	7 (16.7)	17 (26.6)
Overweight	16 (18.4)	16 (27.6)	5 (11.9)	16 (25.0)
Obesity	25 (28.7)	24 (41.4)	26 (61.9)	25 (39.0)
Missing data	9 (10.3)	4 (6.9)	4 (9.5)	6 (9.4)
*Male sex*				
All children	43 (49.4)	37 (63.8)	21 (50.0)	30 (46.9)
Children with overweight	10/16 (62.5)	8/16 (50.0)	2/5 (40.0)	8/16 (50.0)
Children with obesity	8/25 (32.0)	18/24 (75.0)	15/26 (57.7)	13/25 (48.0)
*Age* (*years*)				
All children (*n* = *87*/*58*/*42*/*64*)	9.9 ± 5.1	10.1 ± 4.0	10.6 ± 4.5	10.4 ± 4.2
Children with overweight (*n* = *16*/*16*/*5*/*16*)	12.2 ± 3.6	10.8 ± 2.7	11.7 ± 1.5	9.6 ± 2.8
Girls with overweight (*n* = *6*/*8*/*3*/*8*)	10.6 ± 3.4	10.6 ± 2.0	11.0 ± 1.5	8.7 ± 2.1
Boys with overweight (*n* = *10*/*8*/*2*/*8*)	13.1 ± 3.5	11.1 ± 3.4	12.9 ± 0.2	10.5 ± 3.2
Children with obesity (*n* = *25*/*24*/*26*/*25*)	11.4 ± 5.1	10.3 ± 3.4	10.4 ± 4.1	11.4 ± 3.2
Girls with obesity (*n* = *17*/*6*/*11*/*12*)	11.2 ± 5.0	9.0 ± 1.9	8.7 ± 3.9	10.4 ± 2.8
Boys with obesity (*n* = *8*/*18*/*15*/*13*)	11.9 ± 5.5	10.7 ± 3.8	11.6 ± 3.9	12.3 ± 3.4
*Z-body mass index*				
All children (*n* = *78*/*54*/*37*/*58*)	1.90 ± 2.11	2.80 ± 1.80	3.38 ± 1.97	3.04 ± 1.89
Children with overweight (*n* = *16*/*16*/*5*/*16*)	2.48 ± 0.38	2.33 ± 0.42	2.67 ± 0.66	3.16 ± 1.50
Girls with overweight (*n* = *6*/*8*/*3*/*8*)	2.43 ± 0.44	2.30 ± 0.39	2.32 ± 0.58	3.42 ± 1.86
Boys with overweight (*n* = *10*/*8*/*2*/*8*)	2.51 ± 0.36	2.36 ± 0.48	3.19 ± 0.42	2.90 ± 1.09
Children with obesity (*n* = *25*/*24*/*25*/*25*)	4.27 ± 1.03	4.39 ± 1.23	4.47 ± 0.92	4.24 ± 1.22
Girls with obesity (*n* = *17*/*6*/*11*/*12*)	4.25 ± 0.77	4.71 ± 1.35	4.21 ± 1.02	4.28 ± 0.72
Boys with obesity (*n* = *8*/*18*/*14*/*13*)	4.32 ± 1.52	4.28 ± 1.21	4.67 ± 0.81	4.21 ± 1.58
**Parents**	**n = 68**	**n = 47**	**n = 37**	**n = 59**
*Weight status*				
Underweight	0 (0.0)	0 (0.0)	0 (0.0)	0 (0.0)
Normal	9 (13.2)	5 (10.6)	4 (10.8)	6 (10.2)
Overweight	17 (25.0)	13 (27.7)	10 (27.0)	10 (16.9)
Obesity	37 (54.4)	29 (61.7)	22 (59.5)	40 (67.8)
Missing data	5 (7.4)	0 (0.0)	1 (2.7)	3 (5.1)
*Male sex*				
All parents	28 (41.2)	19 (40.4)	14 (37.8)	25 (42.4)
Parents with overweight	8/17 (47.1)	5/13 (38.5)	4/10 (40.0)	7/10 (70.0)
Parents with obesity	8/37 (21.6)	10/29 (34.5)	8/22 (36.4)	15/40 (37.5)
*Age* (*years*)				
All parents (*n* = *68*/*47*/*37*/*59*)	40.5 ± 7.3	42.6 ± 8.1	43.6 ± 6.1	42.9 ± 6.4
Parents with overweight (*n* = *17*/*13*/*10*/*10*)	44.0 ± 7.2	43.9 ± 5.4	42.6 ± 7.1	42.1 ± 8.6
Women with overweight (*n* = *9*/*8*/*6*/*3*)	42.3 ± 7.6	42.1 ± 5.6	43.4 ± 8.7	42.3 ± 7.2
Men with overweight (*n* = *8*/*5*/*4*/*7*)	45.9 ± 6.7	46.9 ± 3.8	41.2 ± 4.9	41.9 ± 9.6
Parents with obesity (*n* = *37*/*29*/*22*/*40*)	39.4 ± 6.9	42.2 ± 8.5	44.3 ± 5.8	42.9 ± 6.1
Women with obesity (*n* = *29*/*19*/*14*/*25*)	39.0 ± 6.6	40.2 ± 7.7	43.9 ± 6.4	42.2 ± 5.7
Men with obesity (*n* = *8*/*10*/*8*/*15*)	41.1 ± 8.0	46.0 ± 8.9	44.8 ± 5.0	44.1 ± 6.8
*Body mass index* (*kg*/*m^2^*)				
All parents (*n* = *63*/*47*/*36*/*56*)	33.5 ± 8.7	33.0 ± 8.3	32.2 ± 7.1	35.6 ± 9.7
Parents with overweight (*n* = *17*/*13*/*10*/*10*)	27.5 ± 1.5	27.3 ± 1.5	27.6 ± 1.7	28.5 ± 1.1
Women with overweight (*n* = *9*/*8*/*6*/*3*)	27.9 ± 1.3	27.2 ± 1.5	27.6 ± 2.1	28.1 ± 1.6
Men with overweight (*n* = *8*/*5*/*4*/*7*)	27.1 ± 1.6	27.3 ± 1.6	27.7 ± 1.2	28.6 ± 1.0
Parents with obesity (*n* = *37*/*29*/*22*/*40*)	38.7 ± 7.5	37.3 ± 7.6	36.0 ± 6.1	39.2 ± 9.0
Women with obesity (*n* = *29*/*19*/*14*/*25*)	38.4 ± 7.6	37.3 ± 6.4	35.9 ± 6.2	41.9 ± 10.2
Men with obesity (*n* = *8*/*10*/*8*/*15*)	39.9 ± 7.6	37.5 ± 9.8	36.2 ± 6.3	34.8 ± 3.7

**Table 2:** Characteristics of families considered as completers and drop-outers. Data are presented as number of subjects (percentages) or median (1st quartile; 3rd quartile). The data concern families from ProxOb II to IV.

Families	Drop-Outs	Completers	*p*
	*n* = 15	*n* = 81	
*ProxOb*			
II	10 (66.7)	31 (38.3)	0.11
III	2 (13.3)	28 (34.6)	
IV	3 (20.0)	23 (27.1)	
Number of people per family	3 (3; 4)	4 (3; 4)	0.66
Single-parent family	5 (33.3)	51 (63.0)	0.03 *
Number of children per family	2 (1; 3)	2 (1; 3)	0.58
At least one parent per family with overweight/obesity	14/14 (100.0)	77/80 (96.2)	1.00
At least one parent with a precarious economic situation	5/7 (71.4)	39/62 (62.9)	1.00

*p*, statistical *p*-value. *: *p* < 0.05.

## Data Availability

All data generated or analyzed during this study are included in this article. Further enquiries can be directed to the corresponding author.

## Supplementary Materials

The following supporting information can be downloaded at: https://www.mdpi.com/article/10.3390/children9050737/s1, Table S1: Parameters assessed in adults [16,20,21,22,23,24,25,26,27,28,29]; Table S2. Parameters assessed in children [30,31,32,33,34,35,36,37,38,39].
