# Supplementary material for: Designing, Implementing, and Evaluating a Home-Based, Multidisciplinary, Family-Centered Pediatric Obesity Intervention: The ProxOb Program
Source: Children (Basel). 2022 May 17;9(5):737. doi: 10.3390/children9050737 (PMC9139264; doi:10.3390/children9050737)
Supplement: Supplementary file 1 [file children-09-00737-s001.zip › children-1706612-supp.pdf]

Table S1. Parameters assessed in adults.

Parameters	Methods	ProxOb I	ProxOb II	ProxOb III	ProxOb IV	ProxOb V
**Clinical parameters**						
Body weight	Body weight scale (Seca Les Mureaux, France).	T0-T1-T2	T0-T1-T2	T0-T1-T2	T0-T1-T2	T0-T1-T2-T3
Body height	Standing stadiometer, barefoot (Seca Les Mureaux, France).	T0-T1-T2	T0-T1-T2	T0-T1-T2	T0-T1-T2	T0-T1-T2-T3
BMI	Weight / height² (kg/m²) - WHO reference values to determine weight status.	T0-T1-T2	T0-T1-T2	T0-T1-T2	T0-T1-T2	T0-T1-T2-T3
Waist circumference	Measured to the nearest 0.5 cm in a standing position with a standard nonelastic tape that was applied horizontally midway between the last rib and the superior iliac crest.	T0-T1-T2	T0-T1-T2	T0-T1-T2	T0-T1-T2	T0-T1-T2-T3
Body composition	Bioelectrical impedance Tanita MC780 multi frequency segmental body composition analyzer [1]	T0-T1-T2	T0-T1-T2	T0-T1-T2	T0-T1-T2	T0-T1-T2-T3
Blood pressure	SBP and DPB were measured in a seated position using an auditory stethoscope with a blood pressure cuff adapted to the arm circumference (Column Trimline graduated in mmHG, blood pressure cuff Welch Allyn).	T0-T1-T2	T0-T1-T2	T0-T1-T2	T0-T1-T2	T0-T1-T2-T3
**Questionnaires**						
Socio-medico-economic profile	EQ5D 3L [2]		T0-T1-T2	T0-T1-T2	T0-T1-T2	T0-T1-T2-T3
Precarity	EPICES [3]	T0-T1-T2	T0-T1-T2	T0-T1-T2	T0-T1-T2	T0-T1-T2-T3
Quality of life	SF36 [4,5]	T0-T1-T2	T0-T1-T2	T0-T1-T2	T0-T1-T2	T0-T1-T2-T3
Physical activity	Ricci & Gagnon [6], IPAQ [7]	T0-T1-T2	T0-T1-T2	T0-T1-T2	T0-T1-T2	T0-T1-T2-T3
Eating behaviors	Food Frequency and habits questionnaire	T0-T1-T2	T0-T1-T2	T0-T1-T2	T0-T1-T2	T0-T1-T2-T3
Eating profile	TFEQ51 [8]		T0-T1-T2			
	TFEQ-R21 [9]			T0-T1-T2	T0-T1-T2	T0-T1-T2-T3
Sleep habits	Sleep habits and routine questionnaire	T0-T1-T2	T0-T1-T2	T0-T1-T2	T0-T1-T2	T0-T1-T2-T3
Parentality	QAECEP [10,11]			T0-T1-T2	T0-T1-T2	T0-T1-T2-T3

DBP: Diastolic Blood Pressure; EQ5D: EuroQol 5 direction instrument; IPAQ: International Physical Activity Questionnaire; QAECEP: Questionnaire Auto-Évaluation Compétence Éducative Parentale; SBP: Systolic Blood Pressure; SF36: Short Form 36 items; T0: baseline; T1: second evaluation phase; T2: evaluation point after the first 6 months of independence; T3: final evaluation; TFEQ: Three Factors Eating Questionnaire; WHO: World Health Organization.

[1] Verney, J.; Schwartz, C.; Amiche, S.; Pereira, B.; Thivel, D. Comparisons of a Multi-Frequency Bioelectrical Impedance Analysis to the Dual-Energy X-Ray Absorptiometry Scan in Healthy Young Adults Depending on Their Physical Activity Level. *J Hum Kinet*, **2015**, *47*, 73–80. https://doi.org/10.1515/hukin-2015-0063.

[2] Rabin, R.; de Charro, F. EQ-5D: A Measure of Health Status from the EuroQol Group. *Ann Med*, **2001**, *33* (5), 337–343. https://doi.org/10.3109/07853890109002087.

[3] Bihan, H.; Laurent, S.; Sass, C.; Nguyen, G.; Huot, C.; Moulin, J. J.; Guegen, R.; Le Toumelin, P.; Le Clésiau, H.; La Rosa, E.; et al. Association among Individual Deprivation, Glycemic Control, and Diabetes Complications: The EPICES Score. *Diabetes Care*, **2005**, *28* (11), 2680–2685. https://doi.org/10.2337/diacare.28.11.2680.

[4] Ware, J. E.; Sherbourne, C. D. The MOS 36-Item Short-Form Health Survey (SF-36). I. Conceptual Framework and Item Selection. *Med Care*, **1992**, *30* (6), 473–483.

[5] Ware, J.; MA, K.; Keller, S. D. SF-36 Physical and Mental Health Summary Scales: A User's Manual. **2002**, *8*, 23–28.

[6] F. Laureyns & JM. Séné. Questionnaire d'auto-évaluation de l'activité physique d'après Ricci J et Gagnon L, modifié par F. Laureyns et JM. Séné https://onaps.fr/outils-devaluation/ (accessed Nov 29, 2021).

[7] Craig, C. L.; Marshall, A. L.; Sjöström, M.; Bauman, A. E.; Booth, M. L.; Ainsworth, B. E.; Pratt, M.; Ekelund, U.; Yngve, A.; Sallis, J. F.; et al. International Physical Activity Questionnaire: 12-Country Reliability and Validity. *Med Sci Sports Exerc*, **2003**, *35* (8), 1381–1395. https://doi.org/10.1249/01.MSS.0000078924.61453.FB.

[8] Stunkard, A. J.; Messick, S. The Three-Factor Eating Questionnaire to Measure Dietary Restraint, Disinhibition and Hunger. *J Psychosom Res*, **1985**, *29* (1), 71–83. https://doi.org/10.1016/0022-3999(85)90010-8.

[9] Cappelleri, J. C.; Bushmakin, A. G.; Gerber, R. A.; Leidy, N. K.; Sexton, C. C.; Lowe, M. R.; Karlsson, J. Psychometric Analysis of the Three-Factor Eating Questionnaire-R21: Results from a Large Diverse Sample of Obese and Non-Obese Participants. *Int J Obes (Lond)*, **2009**, *33* (6), 611–620. https://doi.org/10.1038/ijo.2009.74.

[10] Gibaud-Wallston, J. Self-Esteem and Situational Stress: Factors Related to Sense of Competence in New Parents, University of Rhode Island, 1977.

[11] Terisse, B.; Trudelle, D. Le Questionnaire d'auto-Évaluation de La Compétence Éducative Parentale (Q.A.E.C.E.P.), 1988.

Table S2. Parameters assessed in children.

Parameters	Methods	ProxOb I	ProxOb II	ProxOb III	ProxOb IV	ProxOb V
**Clinical parameters**						
Body weight	Body weight scale (Seca Les Mureaux, France).	T0-T1-T2	T0-T1-T2	T0-T1-T2	T0-T1-T2	T0-T1-T2-T3
Body height	Standing stadiometer, barefoot (Seca Les Mureaux, France).	T0-T1-T2	T0-T1-T2	T0-T1-T2	T0-T1-T2	T0-T1-T2-T3
z-BMI and BMI percentile	Age and sex specific BMI percentiles for the French population were used to define obesity (97th) [12].	T0-T1-T2	T0-T1-T2	T0-T1-T2	T0-T1-T2	T0-T1-T2-T3
Waist circumference	Measured to the nearest 0.5 cm in a standing position with a standard nonelastic tape that was applied horizontally midway between the last rib and the superior iliac crest.	T0-T1-T2	T0-T1-T2	T0-T1-T2	T0-T1-T2	T0-T1-T2-T3
Body composition	Bioelectrical impedance Tanita MC780 multi frequency segmental body composition analyzer [13,14].	T0-T1-T2	T0-T1-T2	T0-T1-T2	T0-T1-T2	T0-T1-T2-T3
Blood pressure	SBP and DPB were measured in a seated position using an auditory stethoscope with a blood pressure cuff adapted to the arm circumference (Column Trimline graduated in mmHG, blood pressure cuff Welch Allyn).	T0-T1-T2	T0-T1-T2	T0-T1-T2	T0-T1-T2	T0-T1-T2-T3
**Questionnaires**						
Socio-medico-economic profile	EQ5DY [15]					
	10-17 years-old		T0-T1-T2	T0-T1-T2	T0-T1-T2	T0-T1-T2-T3
Quality of life	AUQUEI [16]					
	5-7 years old	T0-T1-T2		T0-T1-T2	T0-T1-T2	
	5-10 years old		T0-T1-T2			
	8-10 years old			T0-T1-T2	T0-T1-T2	
	PESDQL [17]					
	5-7 years old					T0-T1-T2-T3
	8-10 years old	T0-T1-T2				
	8-12 years old					T0-T1-T2-T3
	13-17 years old					T0-T1-T2-T3
	VSPA [18,19]					
	11-17 years old	T0-T1-T2	T0-T1-T2	T0-T1-T2	T0-T1-T2	
Physical activity	Ricci & Gagnon [6]	T0-T1-T2	T0-T1-T2			
	Clinical PA and SB questionnaire [20]			T0-T1-T2	T0-T1-T2	T0-T1-T2-T3
Eating behaviors	Food Frequency and habits questionnaire	T0-T1-T2	T0-T1-T2	T0-T1-T2	T0-T1-T2	T0-T1-T2-T3
Eating profile	CTFEQ-R21 [21]					
	5-10 years old		T0-T1-T2			
	11-14 years old		T0-T1-T2			
	8-14 years old			T0-T1-T2	T0-T1-T2	T0-T1-T2-T3
	15-17 years old			T0-T1-T2	T0-T1-T2	T0-T1-T2-T3
	TFEQ51 [8]					
	15-17 years old		T0-T1-T2			
Sleep habits	Sleep habits and routine questionnaire	T0-T1-T2	T0-T1-T2	T0-T1-T2	T0-T1-T2	T0-T1-T2-T3

AUQUEI: Auto-Questionnaire de Qualité de vie Enfant Imagé; BMI: Body Mass Index; CTFEQ : Children Three Factors Eating Questionnaire; DBP: Diastolic Blood Pressure; EQ5D: EuroQol 5 direction instrument; PESDQL: Pediatric Quality of Life Inventory; SBP: Systolic Blood Pressure; T0: baseline ; T1: second evaluation phase; T2: evaluation point after the first 6 months of independence ; T3: final evaluation; TFEQ: Three Factors Eating Questionnaire; VSPA: Vécu et Santé Perçue de l'Adolescent; WHO: World Health Organization.

[12] Cole, T. J.; Bellizzi, M. C.; Flegal, K. M.; Dietz, W. H. Establishing a Standard Definition for Child Overweight and Obesity Worldwide: International Survey. *BMJ*, **2000**, *320* (7244), 1240–1243. https://doi.org/10.1136/bmj.320.7244.1240.

[13] Verney, J.; Metz, L.; Chaplais, E.; Cardenoux, C.; Pereira, B.; Thivel, D. Bioelectrical Impedance Is an Accurate Method to Assess Body Composition in Obese but Not Severely Obese Adolescents. *Nutr Res*, **2016**, *36* (7), 663–670. https://doi.org/10.1016/j.nutres.2016.04.003.

[14] Thivel, D.; Verney, J.; Miguet, M.; Masurier, J.; Cardenoux, C.; Lambert, C.; Courteix, D.; Metz, L.; Pereira, B. The Accuracy of Bioelectrical Impedance to Track Body Composition Changes Depends on the Degree of Obesity in Adolescents with Obesity. *Nutr Res*, **2018**, *54*, 60–68. https://doi.org/10.1016/j.nutres.2018.04.001.

[15] Kreimeier, S.; Greiner, W. EQ-5D-Y as a Health-Related Quality of Life Instrument for Children and Adolescents: The Instrument's Characteristics, Development, Current Use, and Challenges of Developing Its Value Set. *Value Health*, **2019**, *22* (1), 31–37. https://doi.org/10.1016/j.jval.2018.11.001.
[16] Manificat, S.; Dazord, A.; Cochat, P.; Nicolas, J. [Evaluation of the quality of life in pediatrics: how to collect the point of view of children]. *Arch Pediatr*, **1997**, *4* (12), 1238–1246. https://doi.org/10.1016/s0929-693x(97)82616-4.
[17] Varni, J. W.; Seid, M.; Rode, C. A. The PedsQL: Measurement Model for the Pediatric Quality of Life Inventory. *Med Care*, **1999**, *37* (2), 126–139. https://doi.org/10.1097/00005650-199902000-00003.
[18] Sapin, C.; Simeoni, M.-C.; El Khammar, M.; Antoniotti, S.; Auquier, P. Reliability and Validity of the VSP-A, a Health-Related Quality of Life Instrument for Ill and Healthy Adolescents. *J Adolesc Health*, **2005**, *36* (4), 327–336. https://doi.org/10.1016/j.jadohealth.2004.01.016.
[19] Simeoni, M. C.; Auquier, P.; Antoniotti, S.; Sapin, C.; San Marco, J. L. Validation of a French Health-Related Quality of Life Instrument for Adolescents: The VSP-A. *Qual Life Res*, **2000**, *9* (4), 393–403. https://doi.org/10.1023/a:1008957104322.
[20] Fillon, A.; Masurier, J.; Boirie, Y.; Duclos, M.; Thivel, D. Development of a Adapted Questionnaire to Assess Physical Activity and Sedentary Behaviors among Young Children. *ONAPS' 2020 annual national workshop (Niort, France)*, **2020**.
[21] Bryant, E. J.; Thivel, D.; Chaput, J.-P.; Drapeau, V.; Blundell, J. E.; King, N. A. Development and Validation of the Child Three-Factor Eating Questionnaire (CTFEQr17). *Public Health Nutr*, **2018**, *21* (14), 2558–2567. https://doi.org/10.1017/S1368980018001210.
